# Escalating human–wildlife conflict in the Wolong Nature Reserve, China: A dynamic and paradoxical process

**DOI:** 10.1002/ece3.5299

**Published:** 2019-06-04

**Authors:** Jianying Xu, Jianying Wei, Wenhua Liu

**Affiliations:** ^1^ College of Resource, Environment and Tourism Capital Normal University Beijing China; ^2^ Key Laboratory of Water Cycle and Related Land Surface Process, Institute of Geographic Sciences and Natural Resource Research Chinese Academy of Sciences Beijing China

**Keywords:** compensation, cropland damage mitigation, ecological restoration, human-wild conflic, systematic analysis, wildlife encroachment, Wolong Nature Reserve

## Abstract

Human–wildlife conflict (HWC) has become a conservation focus for both protected area management and local communities in many parts of the world. The incidence and mediation of HWCs are rooted in coupled environmental and socioeconomic contexts. A systematic analysis of HWCs was undertaken in 2016 in the Wolong Nature Reserve located in Sichuan Province, southwestern China. Semi‐structured interviews were conducted with 201 local households to understand the occurrence of wildlife damage, the wildlife species involved, the typical losses incurred, and the mitigation measures employed. The results revealed that local HWC has increased rapidly in recent years due to effective biodiversity conservation and ecological restoration policies. Despite the widespread occurrence of HWCs, with nearly all respondents stating that they had suffered a financial loss, appropriate compensation schemes are lacking. Local respondents' expected compensation amount and style were investigated, and it was concluded that integrated compensation and community development plans are needed to mediate and resolve HWC. In particular, greater attention should be given to reduce local households' dependence on agriculture and transform local livelihood strategies to alternative economic activities not related to farming, such as ecotourism development and migrating employment.

## 1 INTRODUCTION

Human–wildlife conflict (HWC) was brought to global attention in 2003 at the 5th World Park Congress during discussions to address challenges of facing wildlife conservation and protected area management (Madden, 2004). Human–wildlife conflicts develop when the needs and behavior of wildlife negatively impact the goals of humans or when the goals of humans negatively impact the needs of wildlife. Common examples are when wildlife causes crop damage or injure or kill livestock or people. Thus, HWC impacts species conservation and jeopardizes human livelihoods and safety, and its mitigation requires increased resources for natural resource managers (Woodroffe, Lindsey, Romañach, Stein, & ole Ranah, 2005).

In essence, HWC is a feature of landscapes or habitats shared between local communities and wildlife (McLennan & Hill, 2012), where both groups increasingly compete for space and resources (Madden, 2004). In protected area management, the relationship between local communities and biodiversity conservation lies at the heart of many HWC controversies. Because HWCs may complicate the relationship between the local community and biodiversity conservation, the need to alleviate this conflict has become a conservation priority around the globe (Goodrich, Seryodkin, Miquelle, & Bereznuk, 2011; Gurung, Smith, McDougal, Karki, & Barlow, 2008; Hussain, 2000; Inskip & Zimmermann, 2009; Kloskowski, 2011; Liu et al., 2011). Research on the mitigation of HWCs has progressed from wildlife management (Haule, Johnsen, & Maganga, 2002; Thapa, 2010; Woodroffe et al., 2005) to stakeholder analysis (Hemson, Maclennan, Mills, Johnson, & Macdonald, 2009), including investigations of local attitudes (Hemson et al., 2009; Kansky & Knight, 2016; McLennan & Hill, 2012), behaviors (Baruch‐Mordo, Breck, & Broderick, 2009; Jacobs, Vaske, & Roemer, 2012; Madden, 2004), underlying economic incentives (Liu et al., 2011), and even complex conflicts among stakeholders (Madden & McQuinn, 2014). It is evident that HWCs are embedded in complex ecological, social, economic, and political contexts (White et al., 2009; Young et al., 2005), and research should therefore focus not only on the HWC itself but also on its background and dynamics. Systematic analyses of HWCs covering their occurrence, local responses, damages incurred, and compensation mechanisms are needed to inform the design of long‐term conservation strategies, especially in areas where HWCs are incipient and where related management practices have been limited.

China is well‐known for its rich biodiversity, but the country is facing a serious decline in its native species (Liu et al., 2003). The national government has established numerous protected areas, representing more than 15% of Chinese territory (Ministry of Environmental Protection of the People's Republic of China 2015). However, due to a large human population and intensifying encroachment on wildlands as well as the reduction in wild prey densities, HWC in China has escalated, and most incidences occur in or near nature reserves and poor and remote mountainous areas. Li (2011) reported that there were over 6,000 compensation cases for wildlife damages in China countrywide between the late 1990s and the end of 2010. Depredation events on humans, domestic livestock, and crops have resulted in significant financial losses (Cai et al., 2011). In provinces with rich biodiversity resources, including Sichuan, Yunnan, and Guizhou, HWCs are widespread (Liu et al., 2011; Soh et al., 2014). There is growing evidence that HWC is not only a conservation issue but also an issue of serious economic significance in China (Cai et al., 2011; Li, Zhang, & Liu, 2009). However, there is no systematic management scheme in China to address HWC, and the relevant compensation and legal institution are absent (He & Wu, 2010; Zhou et al., 2010). A systematic investigation was undertaken in the paper by conducting a questionnaire survey on people living in the wildlife reserve to find out the extent of and reasons for human–wildlife conflict in order to realize the following purposes: (a) exploring the underlying mechanism of HWC and species responsible for HWC; (b) discussing complicated relationships between biodiversity conservation and HWC or wildlife damage, two contradictory issues in the reserve management; and (c) probing potential measures for mitigating wildlife damage and reconciling the relationship among biodiversity conservation, local livelihoods, and the reserve management. The Wolong Nature Reserve, a reserve famous for Giant Panda (*Ailuropoda melanoleuca*) conservation, was selected as a case‐study site. The results from our analysis of HWCs at this local level could contribute to the development of national‐level policy directives or natural resource conservation legislation.

## 2 MATERIALS AND METHODS

### 2.1 Study area

**Figure 1:**
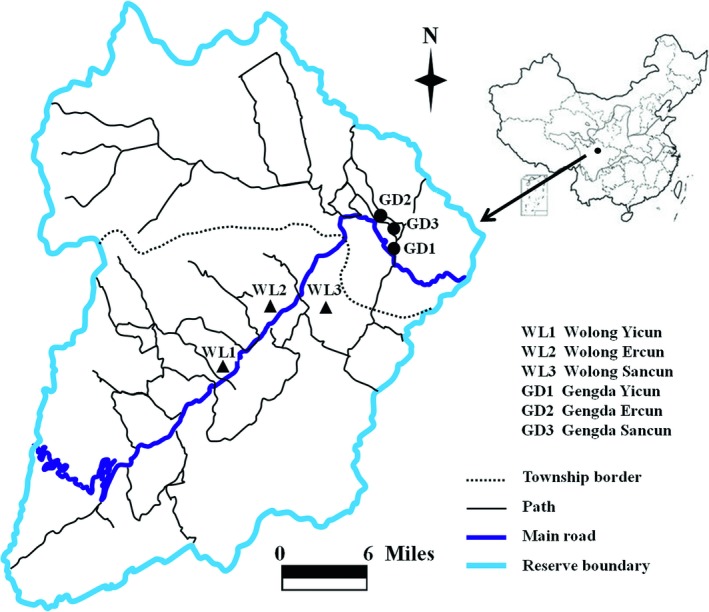
The Wolong Nature Reserve in China and the distribution of local croplands

The Wolong Nature Reserve is the largest of China's 25 nature reserves established to protect the Giant Panda, which is a flagship species for biodiversity conservation in China. There are approximately 100 Giant Pandas that live within the reserve, which represents approximately 10% of the total Panda population in China. The reserve was established in 1963 with an area of 200 km^2^, and it was expanded to its current size of 2,000 km^2^ in 1975 (Liu, Linderman, Ouyang, An, & Zhang, 2001; Liu, Ouyang, Tan, Yang, & Zhang, 1999). The reserve was upgraded to a UNESCO biosphere reserve in 1980. The reserve is located in Wenchuan County, Sichuan Province, southwest China (102°2″ to 103°24″E, 30°45″ to 31°25″N), and is situated in the transition zone from the Chengdu Plain to the Qinghai–Tibet Plateau (Figure 1). The diverse environment of the Wolong Nature Reserve provides a home not only to the Giant Panda but also to 57 other endangered animals and 24 species of rare plants, including the Golden Monkey (*Rhinopithecus roxellanae*) and the Dove Tree (*Davidia involucra*).

The reserve is managed by the Administrative Bureau of Wolong Natural Reserve, under which are two township governments, Wolong Township and Gengda Township. In 2013, there were a total of 1,164 households in the reserve and a population of approximately 5,950 (data provided by the Wolong Nature Reserve Management Bureau). Although Han people are the dominant ethnic group in China, approximately 70% of the people living in the reserve belong to three ethnic minorities: Tibetan, Chang, and Hui. Chengdu–Xiaojin Road is the only main road connecting the local people to the region outside of the reserve.

There are a total of six administrative villages in both townships each with a committee responsible for village management. Each township has jurisdiction over three villages. Wolong Yicun, Wolong Ercun, and Wolong Sancun are included in the Wolong Township. Gengda Yicun, Gengda Ercun, and Gengda Sancun are included in the Gengda Township. Before 2008, four villages (i.e., Gengda Yicun, Gengda Ercun, Wolong Yicun, and Wolong Ercun) were located near the main road, and another two (i.e., Gengda Sancun and Wolong Sancun) were located far from the main road (Figure 1). Following the 2008 Wenchuan earthquake, the two formerly remote villages were relocated to the vicinity of the main road. The relocated people have constructed their houses on croplands owned by villagers who already resided along the main road. The cropland holdings of the four existing villages near the main road have therefore decreased dramatically. The reserve's local villagers depend primarily on traditional agriculture for their subsistence. Corn (*Zea mays*), potato (*Solanum tuberosum*), and cabbage (*Brassica oleracea var. capitata*) are the main crops, of which cabbage is a major source of income. Other local employment opportunities exist but are limited. Such opportunities include housing construction, transportation, collection of medicinal herbs, and forest guarding and patrolling.

In the Wolong Nature Reserve, relationships between the local community and reserve management are complicated (Xu, Chen, Lu, & Fu, 2006). The Giant Panda's suitable habitat has been degraded due to local human activities (Liu et al., 2001). In response, the local reserve management has initiated three ecological restoration programs—the Grain for Green Program (GGP), the Natural Forest Protection Program (NFPP), and the Switch from Fuelwood to Electricity Energy Program. These three forest protection and restoration programs have impacted the local people's lives and livelihoods, and their perception of the reserve management (An, Lupi, Liu, Linderman, & Huang, 2002; Xu et al., 2006). Researchers have devoted significant attention to the Grain for Green Program and its sustainability, land reconversion, and local enrollment (Chen et al., 2012; Chen, Lupi, He, Ouyang, & Liu, 2009; Xu, Chen, Lu, & Fu, 2007), but less attention has been given to the ecological effects of the restoration programs.

### 2.2 Data collection

We interviewed local people to ascertain the occurrence of wildlife damage and the species responsible for the damage (Liu et al., 2009, 2011). The method for this research was approved by institutional review board of Capital Normal University. In our study, a questionnaire was designed to collect relevant information about HWC in the study area. Pretesting was conducted to ensure that respondents could understand the information in the questionnaire, and then, we revised the questionnaire according to their reactions. It had been proved that local cropland damage was significantly related to the location of farmland not its acreage (Xu, Huan, & Kong, 2016). Furtherly, local cropland was allotted to local people equally according to its quality on the village level, of which location was an important feature of quality since it determined accessibility and soil productivity of the cropland. Therefore, we determined the sample size according to local total household number and 20% of local households were planned to be covered in the interview. Since local households are distributed in six villages, the total sample size was also distributed to each village evenly, that is, 20% of household in each village was determined as sample size before interviewing. Then, participating households were selected from each administrative village in the way of convenience sampling, with households recruited based on their availability to participate until the required sample size was reached. Only those who expressed oral consent were interviewed subsequently. Written consent was not obtained due to local predominantly illiterate population in the study area. Respondents were told the purpose and scope of the study, the agency behind it, and how the results would be used at the beginning of interview. Their personal information was recorded in the questionnaire separately but maintained anonymity during analysis and reporting. All the respondents were voluntary, and they could withdraw at any time according to their own will. In each family, one adult (>18 years old) was selected for the interview. Any adult who stated he/she was familiar with human–wildlife conflict in his/her family or study area was selected as respondents. A total of 234 local families participated, but only 201 respondents fulfilled the interview successfully. The other 33 families withdrew the interview due to their unwillingness to continue or something unexpectedly happened such as emergent business.

The interviews were held in October 2016 that was a comprehensive investigation with the questioning covering the occurrence of wildlife damage, the typical damage incurred (e.g., crop raiding, livestock predation, and human injury or death), the common names of the wildlife species causing damage, the households' mitigation measures, any changes in HWC frequency following implementation of the Grain for Green Program, and the respondents' expected compensation for damages incurred. The interviews also included open‐ended questions, to encourage the respondents to discuss their beliefs on the reasons behind any changes in wildlife damage and its spatial distribution. In addition, we also interviewed four local government managers to understand their beliefs about the reasons behind HWC occurrence and the associated policies for conflict mitigation. Among them, two managers come from Administrative Bureau of Wolong Natural Reserve who are in charge of human–wildlife conflicts and confirming wildlife species causing damage. The other two come from Wolong and Gengda governmental agency, respectively, who are in charge of local human litigation from human–wildlife conflict and confirming the loss.

### 2.3 Data analysis

Descriptive analysis in SPSS software is used to present quantitative characteristics of human–wildlife conflict in the study area. All the figures in the paper are finished with EXCEL software in OFFICE 2010. All the data are analyzed and presented at least on town level since local people are under the administration of local township government (Wolong and Gengda township governments), respectively.

## 3 RESULTS

### 3.1 Sociodemographical characteristics of respondents

A total of 201 households were investigated successfully (Table 1). More male respondents were investigated than females. Majority of the respondents were above 30 years old, of which about 44% were aged between 31 and 50 and 53% were above 50 years old. As for education level, more respondents (64%) were educated to primary school or lower, only 8% of the respondents received education higher than senior school.

**Table 1:** Demographic and socioeconomic characteristic of respondents

Characteristics	Wolong			Gengda			Total (%)
	Wolong Yicun	Wolong Ercun	Wolong Sancun	Gengda Yicun	Gengda Ercun	Gengda Sancun	
Number	46	45	19	43	23	25	201 (100)
Gender							
Male	21	26	13	20	11	13	104 (51.7)
Female	25	19	6	23	12	12	97 (48.2)
Age							
≤30	1	3	2	1	0	0	7 (3.5)
31–50	24	21	9	17	9	8	88 (43.8)
≥51	21	21	8	25	14	17	106 (52.7)
Education level							
≤Elementary	33	23	13	28	16	16	129 (64.2)
Junior school	10	16	3	12	6	8	55 (27.4)
Senior school	0	3	2	2	1	1	9 (4.5)
≥College or higher	3	3	1	1	0	0	8 (4.0)
Household size	4.05	4.10	4.00	4.19	4.13	4.33	
Cropland holding per household (Mu)	2.18	1.93	4.52	0.61	2.72	1.04	

### 3.2 Respondents experienced wildlife damage and reasons for HWC

A total of 68% (*n* = 136) of the respondents stated they suffered wildlife damage in 2016 (Table 2). More respondents in Wolong suffered wildlife damage than that in Gengda. In Wolong, 92% (*n* = 101) of respondents stated that they suffered wildlife damage, but in Gengda, about 39% (*n* = 35) of the respondents suffered the damage. At the village level, Wolong Yicun suffered the most serious wildlife damage, and nearly 97.8% (*n* = 45) of the respondents stated they suffered wildlife damage. Also, nearly all respondents in Wolong Sancun (94.7%, *n* = 18) suffered wildlife damage. Respondents in Gengda Yicun suffered the least wildlife damage (20.9%, *n* = 9), followed by Gengda Sancun (40%, *n* = 10).

**Table 2:** Number of respondents experiencing wildlife damage

Responses	Wolong			Gengda			Total
	Wolong Yicun	Wolong Ercun	Wolong Sancun	Gengda Yicun	Gengda Ercun	Gengda Sancun	
Yes	45	38	18	9	16	10	136
No	1	7	1	34	7	15	65

The respondents and government managers described what they perceived were the reasons for HWCs (Table 3). The most commonly mentioned reason was a substantial increase in the number of wild animals (*n* = 164). The second most commonly mentioned reason was environmental improvements resulting from ecological restoration works (*n* = 160), particularly associated with the Grain for Green Program, which had required local people to convert sloping land into forested land. These local people shared the same opinion with the government managers. About 41.3% (*n* = 83) of the respondents attributed HWC to food resource shortage in forest, so local wild animals had to go outside the forest to look for edible food. Neighboring edible crop and livestock became their aims. But also 74% (*n* = 149) of the respondents attributed the HWC to easy ingestion of crops. They thought local main crops, corn and cabbage, are both palatable to and easy ingestion for wild animals.

**Table 3:** Reasons for increasing wildlife damage

Reasons	Wolong	Gengda	Total
Increase in wildlife number for hunting prohibited	92	72	164
Enlargement of home range for ecological restoration	87	73	160
Easy ingestion of crops	85	64	149
Limited forage for wildlife in nature	51	32	83
Change in wildlife feeding habits	13	11	24
Others	2	3	5

### 3.3 Wildlife species causing damage

Although the Wolong Nature Reserve was well known for Giant Panda conservation, it also provided habitat for many other species. In the investigation in 2016, more than 13 wild species, comprising six orders and 11 families, were claimed by local people to be causing damage. Wild boar was the most frequently cited problem species (cited by 40.3% [*n* = 81] of the respondents) followed by Masked civet and Hog badger (cited by 35.8% [*n* = 72] and 29.9% [*n* = 60] of the respondents, respectively; Table 4). The Giant Panda was considered problematic by only 1% (*n* = 2) of the respondents.

**Table 4:** Wildlife species causing damage

Order	Family	Scientific Name	Protection grade/status	Common name	Wolong (%)^a^	Gengda (%)^a^	Total (%)^a^
*Artiodactyla*	*Suidae*	Sus scrofa	TUA	Wild boar	62 (56.4)	19 (20.9)	81 (40.3)
*Carnivora*	*Viverridae*	Paguma larvata	TUA	Masked civet	62 (56.4)	10 (11.0)	72 (35.8)
*Carnivora*	*Mustelidae*	Arctonyx collaris	TUA	Hog badger	38 (34.5)	22 (24.2)	60 (29.9)
*Rodentia*	*Hystricidae*	Hystrix brachyura	TUA	Porcupine	17 (15.5)	8 (8.8)	25 (12.4)
*Primates*	*Cercopithecidae*	To identify	Ⅱ	Monkey	17 (15.5)	0 (0)	17 (8.5)
*Carnivora*	*Canidae*	Canis lupus Linnaeus	Ⅱ	Wolf	10 (9.1)	3 (3.3)	13 (6.5)
*Carnivora*	*Ursidae*	Ursus thibetanus	Ⅱ	Bear	5 (4.5)	0 (0)	5 (2.5)
*Carnivora*	*Felidae*	Panthera pardus	Ⅱ	Leopard	8 (7.3)	1 (1.1)	9 (4.5)
*Mammalia*	*Canidae*	Cuon alpinus	Ⅱ	Dhole	8 (7.3)	0 (0)	8 (4.0)
*Artiodactyla*	*Cervidae*	Rusa unicolor	Ⅱ	Deer	4 (3.6)	2 (2.2)	6(3.0)
*Carnivora*	*Mustelidae*	Mustela sibirica	—	Weasel	4 (3.6)	2 (2.2)	6 (3.0)
*Carnivora*	*Rsidae*	Ailuropoda melanoleuca	Ⅰ	Giant Panda	1 (0.9)	1 (1.1)	2 (1.0)

a Figures outside the brackets represent the number of respondents suffering damage from the species listed. Figures in the bracket represent the percent of respondents suffering damage from the species listed (*N* = 110 for Wolong, *N* = 91 for Gengda, and *N* = 201 for total).

### 3.4 Typical damages and losses

**Figure 2:**
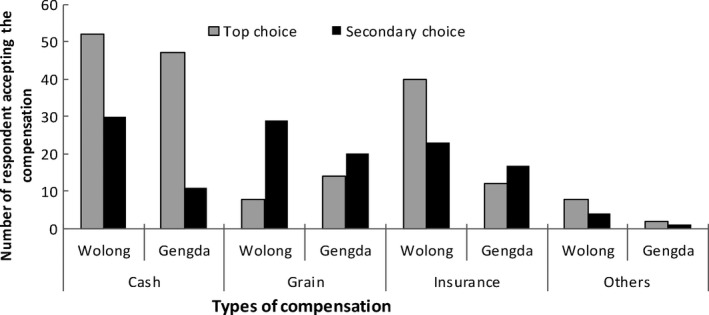
Typical loss and damage in the Wolong Nature Reserve

Crop losses were reported by most respondents as the most frequent and serious damage from HWC (Figure 2). But more respondents in Wolong suffered damage and loss than that in Gengda (86.5% [*n* = 94] in Wolong and 26.4% [*n* = 24] in Gengda). Livestock loss was ranked as the second most serious damage from HWC. About 33.6% (*n* = 37) of the respondents in Wolong and 8.8% (*n* = 8) of the respondents in Gengda suffered livestock loss. Personal injuries caused by wildlife assault on humans happened only once. It was caused by wild ox (*Budorcas taxicolor*) in Wolong.

Various crops and livestock suffered damages to different extents. In the case of crops, almost all crops suffered damage, but plum, corn, and cabbage, which served as the local people's primary crops for income source and pig food, were scavenged most seriously by these wild species (Table 5). Acreage of plum damaged by wildlife reached to 22.5 ha, and acreage of corn and cabbage damaged reached to 7.69 and 7.65 ha, respectively. With respect to livestock loss (Table 6), goat and yak, which was also main income source of local people, suffered the most damage because they were free‐ranging at grassland on the top of mountains. Pigs, the most universal livestock in the study area, received almost no damage because they are reared in pens.

**Table 5:** Wildlife damage on crops

Types of crops	Number of household planting the crops	Number of household damaged by wildlife	Acreage of land planting the crops (ha)	Number of land damaged by wildlife (ha)
Corn	69	54	7.69	1.5
Cabbage	75	41	7.65	1.3
Plum	104	32	22.5	—
Potato	29	17	1.4	0.35
Asparagus lettuce	25	6	2.87	0.23
Radish	13	9	1.5	0.36
Konjak	5	1	0.75	0.01
Others	27	6	0.60	0.1
Total	166	120	44.99	3.9

**Table 6:** Wildlife damage on livestocks

Types of livestock	Number of household breeding the livestock	Number of household damaged by wildlife	Total number of livestock bred by household	Number of livestock damaged by wildlife
Goat	23	14	944	127
Yak	16	8	1,054	45
Chicken	72	21	1,348	92
Cow	32	5	267	16
Pig	116	0	577	0
Total	146	45	4,190	280

### 3.5 Respondents' acceptance of compensation to mitigate HWC

**Figure 3:**
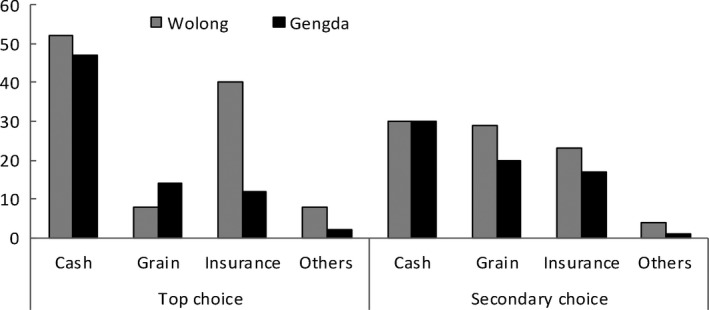
Respondents' acceptance to varied compensation should be placed in line 98, line 226, and line 249, respectively. Legend of Figure 1 is shown in picture

Only 6.0% (*n* = 12) of the respondents once received compensation when Giant Pandas were identified as the culprits of crop damage. Most damage caused by other wildlife was not compensated. Therefore, respondents were asked about their expected compensation to ascertain their acceptance of mitigating measures. We identified four types of compensation used in China or worldwide and asked respondents to select their top choice and secondary choice (Figure 3). Top choice meant respondents' primary choice or the choice they preferred most. Secondary choice meant alternative choices assuming primary choice could not achieve. As for top choice, 99 respondents preferred cash compensation, with 52 respondents in Wolong and 47 respondents in Gengda. Fifty‐two respondents (40 in Wolong and 12 in Gengda) selected commercial insurance as their primary choice. As for their secondary choice, more respondents (*n* = 49) accepted grain compensation than cash compensation (*n* = 41) and commercial insurance (*n* = 40).

Respondents' varied choice disclosed their concerns about the dual characteristics of compensation types. Some respondents preferred cash compensation due to its flexibility and convenience, but some respondents rejected it due to its being restricted to government budgets; grain compensation was accepted by some respondents because they need not to buy grain using compensation cash and declined by some respondents because they worried about the quality of grain being compensated. Commercial insurance was approved by some respondents because they could deal with insurance agent directly instead of dealing with local government, but declined by some respondents because they thought insurance agency was not credible.

In the case of cash compensation amount, respondents claimed their willingness to accept when their crops were damaged. About 76% of the respondents claimed their willingness to accept was between 7,500 and 22,500 Yuan for corn per ha and 71% claimed 15,000–67,500 Yuan for cabbage per ha. Average willingness to accept reached to 19,560 Yuan/ha for corn and 50,970 Yuan/ha for cabbage. Despite the fact that respondents required willingness to accept varied from person to person, most of them referred to actual field yield and market price of crops.

### 3.6 Respondents' current mitigation measures and their acceptance to potential mitigation measures

Almost all of the respondents (92%, *n* = 184) adopted one or more traditional methods to deter wildlife from their cropland. The remaining 8% (*n* = 17) did nothing to protect their crops because they had very little cropland, manpower, or material resources. Among the respondents using traditional methods, approximately 50% (*n* = 100) used field fencing. Although a board fence was the most common style of fencing used, some respondents reported that board fences required significant effort to maintain and were not effective because some animals could simply jump over or burrow under the fence. Additionally, a board fence was considered to have low durability. Forty‐seven percent of the respondents guarded their cropland during the harvesting season. A shack was built near the field for guarding crops at night. Human guarding was most effective against intruding wildlife, but required substantial manpower and time. Setting up a scarecrow was relatively simple, but its effectiveness decreased when animals became familiar with it. Twenty‐two respondents (*n* = 44) often lit fires to scare off wildlife. Additional deterrent methods such as creating loud noise including by setting off firecrackers were also used by approximately 6.5% (*n* = 13) of the respondents.

Potential measures to mitigate the damage, including changing current crop types into crops unpalatable to wild animals, giving up crop planting, and transforming into nonagricultural activities, were provided to respondents and inquired their acceptance (Table 7). Results showed that 14% of the respondents approved changing current crop type into crops more profit but unpalatable to wild animals, such as Chinese herbal medicine. The remainder thought there was no suitable crop which can both substitute current crop and avoid wild animal damage, so refused to take the measure. Only 9% (*n* = 18) of the respondents were willing to give up crop planting. Nearly 70% (*n* = 140) declined the measure because they had no confidence in finding alternative income sources limited by their age, educational attainment, or technology. The remaining respondents (21%, *n* = 43) did not give any opinion. Comparatively, most respondents (56.2%) agreed to transform into nonagricultural activity. Especially, they held a high expectation for local tourism development and were interested in running hotel, restaurant, and shop of local products, etc.

**Table 7:** Respondents' acceptance to potential mitigation measures

Potential measures	Wolong (%)		Gengda (%)		Total (%)	
	Frequency	Percent (%)	Frequency	Percent (%)	Frequency	Percent (%)
Nonagricultural activities						
Agree	79	71.8	34	37.4	113	56.2
Disagree	31	28.1	28	30.8	59	29.3
No opinion	0	0	29	31.9	29	14.4
Giving up planting						
Agree	13	11.8	5	5.5	18	8.9
Disagree	92	83.6	48	52.7	140	69.7
No opinion	5	4.5	38	41.8	43	21.4
Changing crops						
Agree	24	21.8	5	5.5	29	14.4
Disagree	81	73.6	39	42.9	120	59.7
No opinion	5	4.5	47	51.6	52	25.9

## 4 DISCUSSION

### 4.1 Escalating human–wildlife conflict and biodiversity conservation

The results showed that wildlife damage expanded from the remote villages to the villages near the main road. The fact is different from our research in the study area in 2003, when almost all of the wildlife damage occurred in remote villages, for example, Wolong Sancun and Gengda Sancun. Comparing to our research in 2003, a dramatic increase in wildlife damage could be found: Only 10% (*n* = 137) of the respondents reported damage (Xu et al., 2006) in 2003. Besides dramatic increase, there was a striking difference in wildlife damage between Wolong Town and Gengda Town. More wildlife damage happened in Wolong than that in Gengda. The difference might derive from some dissimilarities between two towns. One dissimilarity came from physical conditions. Wolong Town owned more forested land and rugged terrain that local people were hard to access; therefore, more wildlife, including Giant Panda, lived in Wolong than that in Gengda. This was also confirmed by Liu et al research that explored Wolong Town owned more suitable habitat for Giant Panda than Gengda (Liu et al., 2001). The other dissimilarity came from local human disturbance and distribution. Local people in Gengda distributed more widely due to local flat terrain, accordingly local disturbance increased and wildlife damage decreased. It follows that the more the biodiversity resource, the more the wildlife damage, which brought challenges for biodiversity conservation.

In addition, in the study area, increasing HWC has resulted from strict wild species conservation and environmental improvements associated with ecological restoration programs. For biodiversity conservation, local managers have implemented mandatory and severe measures to prohibit hunting and poaching by the local villagers. All of the tools that the villagers used for hunting, such as guns, traps, and snares, were confiscated and banned. All wild animals, not the only Giant Panda, were protected from hunting. This measure has apparently helped the local wildlife populations to increase. The reforested land resulting from Grain for Green Program also broadened forest area and connected natural forest with local cropland like abridge. Thus, wildlife access to cropland became more frequent. Therefore, escalating wildlife damage appears to be an unintended consequence of biodiversity conservation and ecological restoration in the study area. The fact is different from other researches' results in the world, which attributed the persistence and escalation of HWC to shrinking natural habitats and resources for wildlife as humans increasingly encroach on wildlands, expanding human and livestock populations, and increased tourism (Goodrich et al., 2011; Harich, Treydte, Sauerborn, & Owusu, 2013; Hemson et al., 2009; Madden, 2008; Treves & Karanth, 2003). Since HWC is rooted in human–wildlife interactions, it is logical that when one party retreats (i.e., humans), the other party (i.e., wildlife) enters. Thus, biodiversity conservation and ecosystem restoration can help to restore wildlife populations but then also lead to increased HWC (Thirgood & Redpath, 2008; Woodroffe et al., 2005), as has been found elsewhere in China (Li et al., 2009; Liu et al., 2011) and in Ireland (O'Rourke, 2014).

### 4.2 Single compensation and varied species causing damage

Despite ubiquitous wildlife damage, local people rarely obtained appropriate compensation for their losses because compensation was only paid for damages caused by Giant Panda according to local management policy, while damages caused by other local wildlife were neglected. In the case of conservation status of wildlife causing the damage, some wildlives were also listed as conservation species. Table 4 showed that Giant Panda was listed under Grade 1 protection, and six species were listed under Grade 2 protection. According to Chinese Wildlife Conservation Law, Grade 1 and Grade 2 protected species are rare or near‐extinct. Three Uses Animals (TUA), which were considered by the Chinese State Forestry Agency to be beneficial animals with economic and scientific research value, were also among the species perceived to be causing damage. Four species in Table 4 were all Three Uses Animals (TUA). For biodiversity conservation, wildlife species can be classified according to their endangered status; however, for local people there was no difference between the damage caused by the Giant Panda and other wildlife species.

Compensation for damages incurred by HWCs is widely used around the globe not only to help farmers recoup their losses but also to increase their level of tolerance of local wildlife and create a positive human attitude toward nature (Bulte & Rondeau, 2007; Fourli, 1999). But compensation system in China only focuses on damages caused by certain endangered species, including tiger, bear, or elephant (Liu et al., 2011; Soh et al., 2014; Zhang & Wang, 2003), rather than any consideration of actual losses incurred or human welfare. A human loss‐based or human welfare‐based evaluation of wildlife damage is urgently needed for HWC mediation in China.

### 4.3 Mitigation measures

In terms of cropland protection, local people were beset with ineffective measures and unbearable investments of labor and time. They were looking for an acceptable balance between crop losses and their investment. Many respondents actually claimed they would give up their cropland if provided adequate compensation because most croplands damaged by wildlife were located in remote areas with poor soils and low yields. Also, crops planted in remote cropland were mainly corn and potato which were appealing to both wild boar and bear. These crops provide limited cash income for the farmers. The local peoples' interest in remote cropland farming is decreasing due to the intensive manual labor involved in the land's cultivation and the limited cash income it provides. So, there was a great gap between limited cropland income and high costs to mitigate cropland damage. Current spontaneous and isolated local‐level preventive measures to deter wildlife from cropland will be insufficient to mitigate HWC, while diverse measures relating to cropland adjustment, livelihood transformation, and spatial prevention are advised to further mitigate or mediate HWC.

## 5 CONCLUSIONS AND RECOMMENDATIONS

This systematic investigation of HWCs in the Wolong Nature Reserve in China has found that incidences of wildlife damage have increased as a result of rigid biodiversity conservation measures and effective ecological restoration programs. The study therefore highlights a case of wildlife increasingly encroaching on a human‐dominated space as opposed to humans encroaching on wildlife habitat. Local natural resource managers and national‐level policies have focused on endangered wildlife and the damage these species incur, but have ignored the sizable increases in common wildlife and the subsequent effects on the local community. Endangered wildlife, common wildlife, and local communities coexist in an ecosystem and should therefore all be equally considered in a holistic approach to ecosystem management. Regulations outlining compensation based on actual damages incurred rather than the species of wildlife causing the damage should be promptly established in the Wolong Nature Reserve and more broadly throughout China.

Natural resource managers should focus not only on biodiversity conservation and ecological rehabilitation but also on the potential consequences for human–wildlife interactions. Managers in the Wolong Nature Reserve were faced with protecting wild animals (including rare and near‐extinct species) on the one hand and protecting local people and their croplands on the other. Our study results might be helpful for making local mitigation measures. Firstly, financial compensation based on human loss should be taken as soon as possible. In addition, we advised that the amount of compensation should be based on market value or yield of crops which was more fair and easily accepted by local people. Secondly, since Chinese law now bans wildlife hunting and indiscriminate killing, cropland adjustment and livelihood transformation of local people are other means of mitigating HWCs in addition to financial compensation. Changing local livelihood strategies and transferring local attention to better‐paying alternatives, such as ecotourism, should be encouraged to fill the gap between limited cropland income and high costs to mitigate cropland damage. Thirdly, it was notable that wildlife damage did not occur evenly in Wolong Town and Gengda Town but more intensively occurred in Wolong Town. Furtherly, it was also obvious that crops planted in remote cropland were mainly corn and potato which were appealing to wild boar, so was the livestock free‐ranging on the mountains to carnivores. Thus, we need to strengthen the study of wildlife behavior and feeding habits so as to distinguish the landscape or habitat shared by local people and wildlife and establish spatial prevention measures. Planting of crops that are unpalatable to wild animals in remote area is also advisable for local people.

## CONFLICT OF INTEREST

None declared.

## AUTHOR CONTRIBUTION

Dr Jianying Xu contributed to data collection, designed the study, and wrote the manuscript. Ms Jianying Wei worked in field survey and collected the data. Dr. Wenhua Liu designed and revised the manuscript.

## ETHICAL APPROVAL

All procedures performed in studies involving human participants were in accordance with the ethical standards of the institutional and/or national research committee and with the 1964 Helsinki declaration and its later amendments or comparable ethical standards.

## INFORMED CONSENT

Informed consent was obtained from all individual participants included in the study.

## Data Availability

All authors agreed to deposit data from this manuscript to public repository. Data are submitted to Dryad, and DOI number is https://doi.org/10.5061/dryad.185q88f.
