# Supplementary material for: Escalating human–wildlife conflict in the Wolong Nature Reserve, China: A dynamic and paradoxical process
Source: Ecol Evol. 2019 Jun 4;9(12):7273–83. doi: 10.1002/ece3.5299 (PMC6662261; doi:10.1002/ece3.5299)
Supplement: Supplementary file 1 [file ECE3-9-7273-s001.docx]

**Appendix**

**Human–wildlife conflict investigation in the Wolong Nature Reserve**

**Questionnaire No**.：_________; **Date**：_______; **Location(Village)**:_________

**Respondents’ Characteristics**:

Age________; Gender_________; Education level______; Occupation_________.

**Household socioeconomic status**

Family size:______; Number of room:_______; Household income_________.

**Wildlife damage investigation**

1、Are you affected by wildlife damage in 2016? (1) Yes (2) No

If yes, please list the common name of the species _____、______、_______、______、______．

2、What aspects of your family are affected by wildlife?

（1）Personal injury

（2）Crops

Types and acreage of cropland: type 1_____, acreage of planted____; acreage of being damaged____; species causing damage_____

Types and acreage of cropland: type 2_____, acreage of planted____; acreage of being damaged____; species causing damage_____

Types and acreage of cropland: type 3_____, acreage of planted____; acreage of being damaged____; species causing damage_____

（3）Livestock

Species and number of Livestock raising: type 1_____, number of livestock raised___; number of livestock damaged_____; species causing damage____;

Species and number of Livestock raising: type 2_____, number of livestock raised___; number of livestock damaged_____; species causing damage____;

Species and number of Livestock raising: type 3____, number of livestock raised___; number of livestock damaged_____; species causing damage____;

（4）Others

3、What are the effects of wildlife on your family?

（1）Personal safety is at stake

（2）Income from agriculture become uncertain

（3）Others______

4、How many and what extent does wildlife affect your crops?

(1) Heavily (2)General (3) A little (4) No

Acreage of cropland damaged by wildlife______

5、How many and what extent does wildlife affect your livestock?

(1) Heavily (2)General (3) A little (4) No

Number of livestock damaged by wildlife______

6、What extent does wildlife affect your home life?

(1) Heavily (2)General (3) A little (4) No

7、Which of the following periods do you think home life were more damaged by wildlife?

（1）Before establishment of Wolong Nature Reserve

（2）After establishment of Wolong Nature Reserve

（3）No change before and after establishment of Wolong Nature Reserve

（4）Before Grain for Green Project

（5）After Grain for Green Project

（6）No changes before and after Grain for Green Project

8、Which of the following periods do you think crop is more severely damaged by wildlife?

（1）Before establishment of Wolong Nature Reserve

（2）After establishment of Wolong Nature Reserve

（3）No change before and after establishment of Wolong Nature Reserve

（4）Before Grain for Green Project

（5）After Grain for Green Project

（6）No changes before and after Grain for Green Project

9、Which of the following periods do you think livestock suffered more damage from wildlife?

（（1）Before establishment of Wolong Nature Reserve

（2）After establishment of Wolong Nature Reserve

（3）No change before and after establishment of Wolong Nature Reserve

（4）Before Grain for Green Project

（5）After Grain for Green Project

（6）No changes before and after Grain for Green Project

10、What do you think is the main cause of frequent wildlife damage?

(1) Hunting is banned and the number of wild animals is increasing

(2) Ecological environment is improved and the range of wildlife activities are expanded

(3) There is insufficient food for wild animals

(4) There are changes in feeding habits of wild animals

(5) Crops are more accessible

(6) Others

11、Have you taken measures to prevent wildlife damage?（1）Yes （2）No

If yes，please list your measures and their effectiveness______

Do you have some other measures to suggest？______

**Investigation on compensation for wildlife damage**

12. Do you receive compensation for wildlife damage?(1)Yes (2) No

If yes，the current compensation method and amount respectively are _________

13 Are you satisfied with the way of compensation? (1) Satisfied (2) Dissatisfied

The reasons are ____________________

14 Are you satisfied with this compensation amount? (1) Satisfied (2) Dissatisfied

If no, the minimum compensation you are willing to accept is ______

15. Do you think compensation should be given for damages caused by animals not listed in the national and local protection list, such as wild boar?(1) Yes (2) No

If yes, they are _____________

16. Do you think the compensation mitigate the conflict between the giant panda and the local residents?

(1) Yes (2) No

Reasons are _____________

17、There are some potential compensation styles，please select and sort them according to your willing．Your top choice is_____________, your secondary choice is _____________

(1) Cash compensation

(2) Grain compensation

(3) Commercial insurance

(4) Other compensation types such as a fencing subsidy

18. Who do you think should be responsible for and compensate for the damage caused by wildlife?

(1) It is a natural disaster, which can only be borne by oneself.

(2) Wildlife belongs to everyone and everyone should be responsible

(3) Wildlife is owned by the state and it’s the responsibility of the central government

(4) Involved departments of local government

(5) Local protection authority

(6) Others

19 There are some potential mitigation measure，please select and give explanation according to your willing．

(1) Changing current crop types into crops unpalatable to wild animals. (agree, neutral, disagree)

Reasons:

(2) Giving up crop planting (agree, neutral, disagree)

Reasons:

(3) Transforming into nonagricultural activities (agree, neutral, disagree)

Reasons:

20. Your Suggestions and opinions on current compensation measures for wildlife accidents.

＿＿＿＿＿＿＿＿＿＿＿＿＿＿＿＿＿＿＿＿＿＿＿＿＿＿＿＿＿＿＿＿＿＿＿＿＿＿＿＿＿＿＿＿＿＿＿＿＿＿＿＿＿＿＿＿＿＿＿＿＿＿＿＿＿＿＿＿＿＿＿＿＿＿＿＿＿＿＿＿＿＿＿＿＿＿＿＿＿＿＿＿＿＿＿
